# Examining the Role of Mitochondria in Ca^2+^ Signaling in Native Vascular Smooth Muscle

**DOI:** 10.1111/micc.12039

**Published:** 2013-05-10

**Authors:** John G McCarron, Marnie L Olson, Calum Wilson, Mairi E Sandison, Susan Chalmers

**Affiliations:** * Strathclyde Institute of Pharmacy and Biomedical Sciences, University of StrathclydeGlasgow, UK; † Department of Biomedical Engineering, University of StrathclydeGlasgow, UK

**Keywords:** Smooth muscle, mitochondria, calcium signalling, imaging

## Abstract

Mitochondrial Ca^2+^ uptake contributes important feedback controls to limit the time course of Ca^2+^signals. Mitochondria regulate cytosolic [Ca^2+^] over an exceptional breath of concentrations (∼200 nM to >10 μM) to provide a wide dynamic range in the control of Ca^2+^ signals. Ca^2+^ uptake is achieved by passing the ion down the electrochemical gradient, across the inner mitochondria membrane, which itself arises from the export of protons. The proton export process is efficient and on average there are less than three protons free within the mitochondrial matrix. To study mitochondrial function, the most common approaches are to alter the proton gradient and to measure the electrochemical gradient. However, drugs which alter the mitochondrial proton gradient may have substantial off target effects that necessitate careful consideration when interpreting their effect on Ca^2+^ signals. Measurement of the mitochondrial electrochemical gradient is most often performed using membrane potential sensitive fluorophores. However, the signals arising from these fluorophores have a complex relationship with the electrochemical gradient and are altered by changes in plasma membrane potential. Care is again needed in interpreting results. This review provides a brief description of some of the methods commonly used to alter and measure mitochondrial contribution to Ca^2+^ signaling in native smooth muscle.

## Fine Control of Ca^2+^ Signaling and Biological Responses

Changes in the [Ca^2+^]_c_ trigger numerous vascular smooth muscle cell activities, which include cell division, growth, metabolism, contraction, and death. To enable the ion to modulate such a diversity of activities, there are a wealth of different types of Ca^2+^ signals (the Ca^2+^ toolkit, 6,45) with various amplitudes, durations, and frequencies and the signal may be confined to particular parts of the cell. Each of these features (amplitude, frequency, duration, location) may selectively target Ca^2+^ signals to particular physiological responses. A central requirement for the existence of the various temporal and spatial signals are local feedback processes which shape the Ca^2+^ increase or restrict changes in the [Ca^2+^]_c_ to small parts of the cell. Mitochondria are of acknowledged significance in the feedback control of Ca^2+^ signaling 15. The organelle's facility for rapid Ca^2+^ uptake controls the Ca^2+^ signal and the altered signal is transduced to a biological response by mitochondria themselves or by other parts of the cell.

The two main sources of Ca^2+^ are the extracellular fluid and the intracellular stores (the SR). Ca^2+^ enters the cell from the extracellular fluid via channels on the plasma membrane such as the voltage-dependent and store-operated Ca^2+^ channels. The other main Ca^2+^ source is the SR store from which release proceeds via two receptor-controlled channels—the IP_3_R and the RyR 8,44. Significantly, the sources of Ca^2+^ are not independent; Ca^2+^ influx regulates Ca^2+^ release and Ca^2+^ release regulates Ca^2+^ influx. For example, Ca^2+^ release from the SR may alter plasma membrane ion channel activity to regulate the membrane potential and Ca^2+^ entry, whereas depletion of the SR of Ca^2+^ activates influx via store-operated Ca^2+^ channels 5,47,53,80. Thus, a change in [Ca^2+^]_c_ arising from the activity of channels in the plasma membrane or the SR will itself regulate ion channel activity to provide feedback control of Ca^2+^ signals.

The strategic positioning of channels, receptors, and organelles is important in facilitating the operation of feedback processes. Various channels, receptors, and organelles combine to become functional units and enable Ca^2+^ to act as either a highly localized signal or to evoke more widespread effects through the cell. For example, in sympathetic neurons, while muscarinic and bradykinin receptors each stimulate PLC to produce IP_3_, only bradykinin receptors co-immunoprecipitate with, and activate, IP_3_R to evoke Ca^2+^ release 19. The arrangement enables different responses to be evoked depending on whether PLC is activated by muscarinic or bradykinin receptors; muscarinic receptors play a key role in regulating neuronal excitability 10 and bradykinin receptors mediate inflammation and hyperalgesia 20.

Active IP_3_R are positioned near the plasma membrane providing another mechanism for agonists, acting via IP_3_, to target specific cellular responses by generating Ca^2+^ rises in specific regions of the cell 74. For example, in cerebral arteries endothelial membrane projections extend through the internal elastic lamina to adjacent smooth muscle membranes. In the projections, local IP_3_-mediated Ca^2+^ release events (referred to as “pulsars”) activate intermediate conductance, Ca^2+^-sensitive potassium channels, which co-localize to the same region, to hyperpolarize the endothelial membrane. The resultant membrane potential change is transmitted from the endothelium to the smooth muscle cells by coupling of the membrane via the projections. In this way localized IP_3_-mediated Ca^2+^ release in endothelial cells triggers relaxation in smooth muscle cells 40. RyR are also organized to contribute to feedback activity and may be coupled to channels on the plasma membrane to form functional units. Local Ca^2+^ release events from RyR (Ca^2+^ sparks), may activate either Ca^2+^-activated K^+^ channels or Ca^2+^-activated Cl^−^ channels or both to generate spontaneous transient outward (hyperpolarizing) or inward (depolarizing) currents on the plasma membrane 4,63,80,81. This facility again permits local Ca^2+^ release via RyR to activate or inhibit smooth muscle function. Another structural element to the organization of Ca^2+^ signals lies in the clustering surface receptors in certain regions on the plasma membrane. The clustering of surface receptors provides areas with increased sensitivity to extracellular stimuli 77 that contribute to feedback control and achieves local specificity in Ca^2+^ signaling.

**Figure 1:**
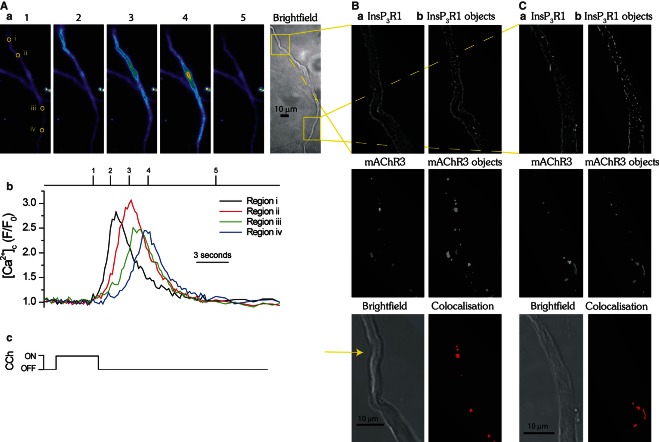
The colocalization of InsP_3_R1 and mAChR3 at sites of Ca^2+^ wave initiation. Carbachol (CCh, 4 s; **A**c) evoked a Ca^2+^ wave (**A**a, **A**b) which initiated from a single site in a single colonic myocyte (Aa; frame 1, region i) and propagated from there. The Ca^2+^ wave repeatedly initiated from the same site during subsequent carbachol applications (data not shown). The extent of IP_3_R1 and mAChR3 colocalization was next assessed in the same cell at the site of Ca^2+^ wave initiation and compared to another separate region of the cell (Aa; yellow boxes on bright field image). The cell was fixed, prepared for immunocytochemistry, labeled with IP_3_R1 monoclonal and mAChR3 polyclonal antibodies, and visualized by confocal microscopy using a fluorescently conjugated secondary antibody (**B**a, **C**a; top and middle panels). Colocalization was quantified using image analysis software ImageJ 65 and the plugin JACoP to examine object-based colocalization. Colocalization of the center of mass of three dimensional IP_3_R1 and mAChR3 objects created using the 3D object counter plugin (**B**b, **C**b; top and middle panels) were quantified by determining the number of centers from one image that were colocalized with objects from the other image (**B**b, **C**b; bottom panel). In the above experiment, 9.0% of the objects colocalized (9 out of 100 objects detected) at the initiation site and 4.6% colocalized (7 out of 152 objects detected) at the other site. The images shown are from plane 45 of 70 (B; initiation site) and plane 34 of 50 (C; same cell, other site) plane z stacks, each image taken at 150 nm intervals. Brightfield image (Ba, Ca; lower panel) of the same cell; initiation site indicated (Ba; yellow arrow). Note: Scale bar at bottom of bright field images. The images acquired using confocal microscopy (B,C) were rotated for clarity to match the orientation of the images acquired using epifluorescence microscopy (A). From Olson *et al*. 60 with permission.

Regenerative propagation of the Ca^2+^ rise (a “Ca^2+^ wave”) occurs by positive feedback control of the Ca^2+^ rise and is another example of interaction among components, in this case to increase the reach of a local Ca^2+^ signal. In smooth muscle, local Ca^2+^ release from the SR may activate neighboring closely positioned ion channels on the SR to propagate the signal from site to site through the cell 9,32,42,46,50 in a way reminiscent of a “fire-beacon” relay network. An interesting feature of waves in smooth muscle is that the Ca^2+^ rise begins at precisely the same small site on each activation despite the cell being stimulated uniformly across the plasma membrane, indicating that is there is a preferred site of wave initiation 60. That local precision in wave initiation is also explained by receptor complexes, in this case which contain mAChR3 and IP_3_R1 that are structurally and functionally coupled (Figure 1) 60. mAChR3 and IP_3_R co-localize to lie within 40–100 nm of each other to generate junctions which facilitate a privileged delivery of IP_3_ to particular IP_3_R 60. This arrangement circumvents the diffuse global signaling that is normally associated with IP_3_ and the inositide acts as a targeted, highly localized signal. Specific associations between signaling proteins located at the plasma membrane (e.g., mAChR3) and the SR membrane (IP_3_R) is one element of feedback control in the Ca^2+^ toolkit enabling receptors to generate differences in the signaling pathways subsequently activated 28,79.

## Role of Mitochondria in Ca^2+^ Signaling

In addition to the feedback arising from the positioning of channels and receptors, intracellular organelles may also regulate Ca^2+^ signals to contribute to the Ca^2+^ toolkit. Mitochondria may take up and sequester a large amount of Ca^2+^ from the cytoplasm and modulate the time course and amplitude of Ca^2+^ signals and shape the resulting message. The mitochondrial capacity to sequester Ca^2+^ is enormous and the buffer power [100,000; 14] is three orders of magnitude greater than that of cytoplasm [∼100; 35]. The mitochondrial buffer power arises largely from the quantities of phosphate within the organelle (∼5 mM). After uptake, Ca^2+^ is slowly exported from mitochondria via a Na^+^-(or H^+^-) Ca^2+^ antiporter mechanism 13.

Mitochondria regulate numerous Ca^2+^ signals including those arising from Ca^2+^ release via IP_3_R or RyR or voltage-dependent Ca^2+^ entry across the outside membrane. Interestingly, mitochondrial Ca^2+^ uptake may decrease or increase the amplitude of Ca^2+^ signals. In some cells, mitochondrial Ca^2+^ uptake decreases the amplitude of IP_3_-evoked Ca^2+^ signals; preventing the organelle from taking up Ca^2+^ increases IP_3_-mediated Ca^2+^ release in cultured hepatocytes and HeLa cells and Ca^2+^ wave velocity in cultured astrocytes 1,7,26. In other cells (smooth muscle, astrocytes and HeLa cells) mitochondrial Ca^2+^ uptake facilitates IP_3_-evoked Ca^2+^ release so that preventing mitochondria from taking up Ca^2+^ reduces the amplitude of the Ca^2+^ signal 13,16,23,31,59,73 (see below).

**Figure 2:**
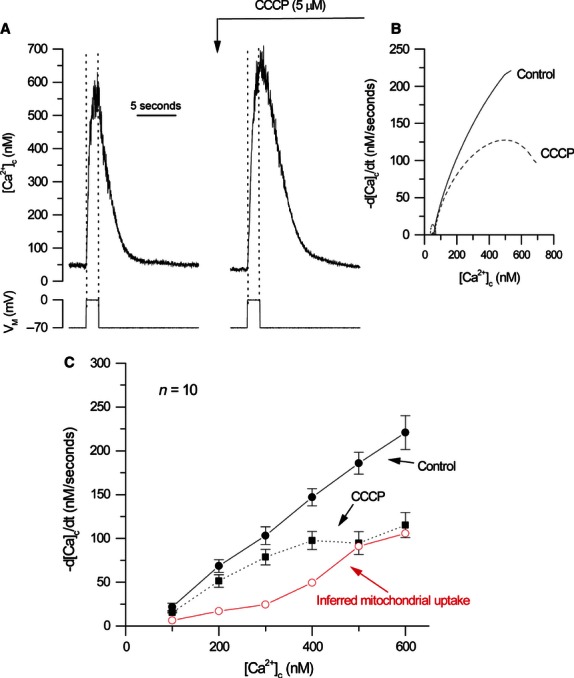
Mitochondria contribute to [Ca^2+^]_c_ decline following voltage-dependent Ca^2+^ entry in smooth muscle. (**A**) Depolarization (−70 to 0 mV) activated a voltage-dependent Ca^2+^ current (data not shown) and increased [Ca^2+^]_c_ in a single colonic myocyte. CCCP (5 μM) slowed the rate of decline of [Ca^2+^]_c_ on repolarization compared with control. (**B**) The rate of decline (−d[Ca^2+^]_c_/d*t*), obtained from high order polynomial fits to the declining phase of the transients, shows a significant slowing when mitochondria were prevented from accumulating Ca^2+^. (**C**) A summary of the rates of decline for 10 cells in the presence and absence of CCCP. The inferred mitochondrial contribution to the decline of [Ca^2+^]_c_ (red line) was obtained by subtracting control rates from those seen in CCCP and shows that mitochondrial Ca^2+^ uptake occurred above 200 nM [Ca^2+^]_c_ (from McCarron & Muir 49 with permission).

Ca^2+^ signals from RyR activity may also be regulated by mitochondrial Ca^2+^ uptake. Preventing mitochondria from taking up Ca^2+^ prolonged caffeine-evoked [Ca^2+^]_c_ increases in aortic and arterial myocytes 25,36. However, in other studies, preventing mitochondrial Ca^2+^ uptake did not alter caffeine-evoked SR Ca^2+^ release in cardiac or various smooth muscles 38,72,75,78. The Ca^2+^ transient arising from voltage-dependent Ca^2+^ entry has an accelerated rate of decline as a consequence of mitochondrial Ca^2+^ uptake and when the uptake is inhibited, the rate of Ca^2+^ decline is substantially slowed (Figure 2) 21,27,49,52.

**Figure 3:**
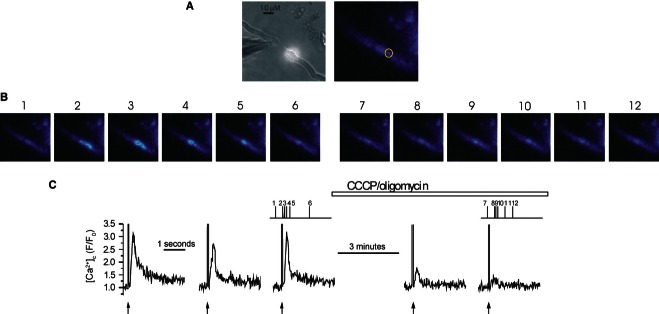
Depolarization of the mitochondrial membrane potential with CCCP inhibits Ca^2+^ release from an IP_3_R cluster (Ca^2+^ puffs). At −70 mV, locally photolyzed caged IP_3_ (25 μM) (↑, C) in a ∼20 μm diameter region (**A**; bright spot in left hand panel, see also whole cell electrode, left side) evoked Ca^2+^ puffs in a single colonic smooth muscle cell (**B**, **C**). There were two individual Ca^2+^ puff sites activated by photorelease of IP_3_. Flash photolysis of IP_3_ every ∼60 s generated approximately comparable [Ca^2+^]_c_ increases (C). Superfusion of CCCP (applied with oligomycin; 1 and 6 μM, respectively) while continuing to photolyze IP_3_ at ∼60 intervals, decreased the amplitude of IP_3_-mediated Ca^2+^ puffs (B, C). The [Ca^2+^]_c_ images (B) are derived from the time points indicated by the corresponding numbers in (C). [Ca^2+^]_c_ changes in (B) are expressed by color; dark blue low and light blue high [Ca^2+^]_c_. Measurements were made from a 3 × 3 pixel box (A; right hand panel, white square). The large increase in fluorescence (C) at the time of photolysis (↑) is artifact from the flash lamp (from Olson *et al*. 59 with permission).

In controlling Ca^2+^ signals, mitochondria operate over a very wide [Ca^2+^]_c_ range (200 nM to >10 μM) 51,64,76. In the example shown in Figure 2, mitochondria modulate signals over the [Ca^2+^]_c_ range 200–600 nM, which demonstrates that mitochondria have a high affinity for Ca^2+^ (in the sub-micromolar range; Figure 2; [see also 64,75]). Interestingly, mitochondria do not appear to alter the rate of rise of the Ca^2+^ transient suggesting that the organelle does not modulate the high local [Ca^2+^] (>10 μM) near active voltage-dependent Ca^2+^ channels (Figure 2) or the activity of the channels themselves. However, mitochondria do have the capacity to modulate Ca^2+^ signals that are ∼2 orders of magnitude larger than the global Ca^2+^ transient from voltage-dependent Ca^2+^ channels and in the tens of micromolar range 67. One notable example in smooth muscle is mitochondrial regulation of the Ca^2+^ signals which arise from the activity of a single IP_3_R cluster (“Ca^2+^ puffs”; Figure 3). When mitochondria are prevented from taking up Ca^2+^ (using uncouplers, complex I inhibitors or uniporter inhibitors), Ca^2+^ puffs are inhibited 59. This observation suggests the organelle may also have a very low affinity for Ca^2+^ (as Ca^2+^ puffs are >10 μM) and that Ca^2+^ uptake is fast enough to modulate concentrations near ion channels.

**Figure 4:**
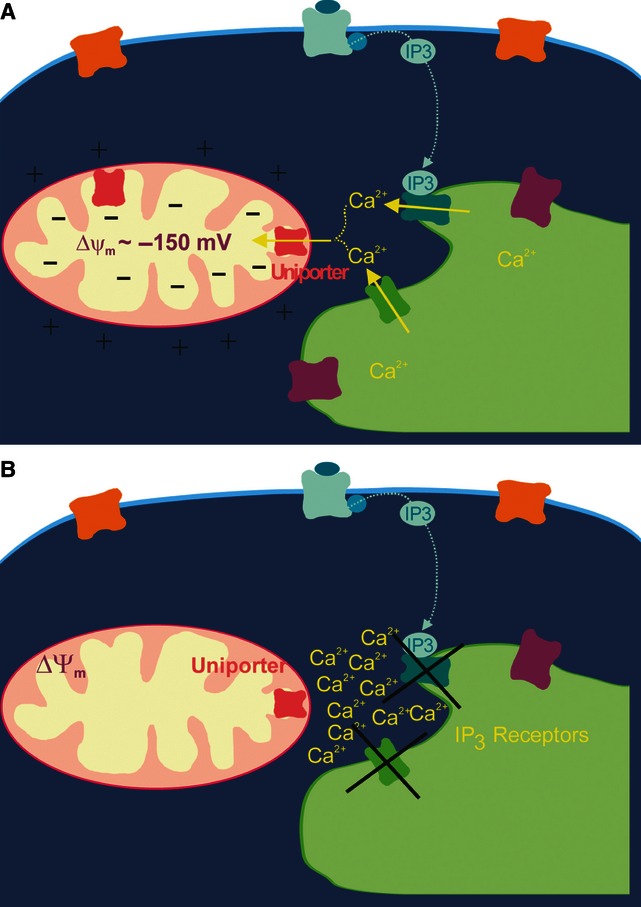
Depolarization of the mitochondrial membrane potential with CCCP inhibits IP_3_-evoked Ca^2+^ release. (**A**) Mitochondria, by buffering the Ca^2+^ rise at IP_3_R, prevents a Ca^2+^-dependent persistent deactivation of IP_3_R to maintain the overall release of Ca^2+^ from the SR. (**B**) When mitochondria are prevented from taking up Ca^2+^ (using uncouplers, complex I inhibitors or uniporter inhibitors) there is a local increase in [Ca^2+^] at IP_3_R promoting Ca^2+^-dependent negative feedback inhibition of IP_3_R activity and preventing Ca^2+^ release.

The question arises, how do mitochondria, by removing Ca^2+^ from the cytoplasm (i.e., lowering [Ca^2+^]), generate a larger [Ca^2+^]_c_ rise? IP_3_R is regulated by Ca^2+^-dependent positive and negative feedback mechanisms. Mitochondrial Ca^2+^ uptake limits a negative feedback inhibition of Ca^2+^ on IP_3_R. There are at least two types of Ca^2+^-dependent negative feedback mechanisms, which may deactivate smooth muscle IP_3_R. In the first, a Ca^2+^-dependent deactivation of IP_3_R occurs at [Ca^2+^]_c_ which exceed ∼300 nM 29. The onset is rapid and the deactivation persists for ∼5 s after the [Ca^2+^]_c_ increase ends in permeabilized vascular smooth muscle 30. Another form of Ca^2+^-dependent deactivation of IP_3_R, once initiated by an increased [Ca^2+^]_c_, persisted long (tens of seconds) after [Ca^2+^]_c_ had regained resting values, that is became, at least partially, refractory 48,58. Each of these processes would persistently inhibit Ca^2+^ release via IP_3_R. Mitochondrial Ca^2+^ uptake by buffering the Ca^2+^ rise at IP_3_R presumably prevents a persistent deactivation of IP_3_R to increase the overall release of Ca^2+^ (Figure 4).

**Figure 5:**
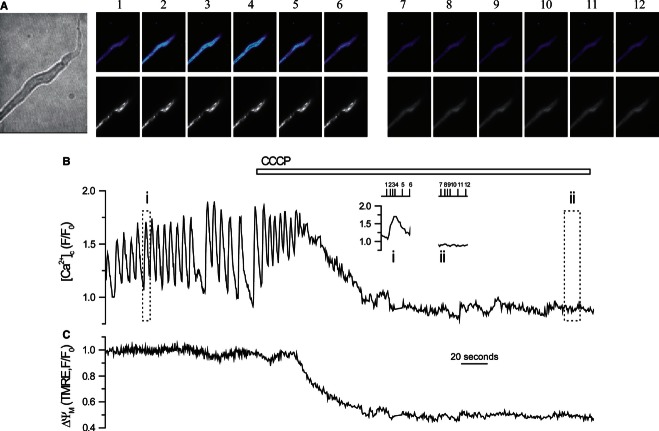
Depolarization of the mitochondrial membrane potential with CCCP blocks Ca^2+^ oscillations. Ca^2+^ oscillations measured with fluo-4 (**A)** upper panel and (**B**) in a single portal vein smooth muscle cell (A left panel) were inhibited by CCCP (applied with oligomycin; 1 and 6 μM, respectively; B open bar). CCCP depolarized ΔΨ_M_ (A lower panel, **C**) as measured by TMRE fluorescence changes and shown as the decrease in fluorescence ratio (C). This suggests that mitochondrial Ca^2+^ uptake is required for Ca^2+^ oscillations to occur (see text). The [Ca^2+^]_c_ images (A) are derived from the time points indicated by the corresponding numbers in (B). The insets (B i,ii) are Ca^2+^ responses in boxes i,ii shown on an expanded time base. [Ca^2+^]_c_ changes in (A, upper panels) are expressed by color; dark blue low and light blue high [Ca^2+^]_c_. ΔΨ_M_ (A, lower panels) is shown as punctate white staining, which decreases with ΔΨ_M_ depolarization. Frames 1–6 (A) are controls (before CCCP/oligomycin) and frame 7–12 after CCCP/oligomycin.

The control that mitochondria exert on Ca^2+^ puffs enables the organelle to exert particularly dramatic effects on Ca^2+^ waves and repetitive Ca^2+^ rises (oscillations). When mitochondria are prevented from taking up Ca^2+^, waves and oscillations halt (Figure 5), that is mitochondrial control extends beyond modulation of the time course of a Ca^2+^ rise and the organelle determines whether some signals (waves and oscillations) occur at all.

In some cell types, mitochondria act as a “firewall” and prevent the Ca^2+^ signal from reaching certain regions of the cell. In pancreatic acinar cells, mitochondria are arranged as a “belt” and prevent Ca^2+^ signals from entering the basal part of the cell 62. However, in smooth muscle, mitochondria do not normally appear to prevent the Ca^2+^ wave from progressing through the cell, but rather the organelle performs a decision-making role to determine whether or not the signal is permitted to progress. If the ΔΨ_M_ is normal and polarized, the Ca^2+^ signal progresses. On the other hand, if ΔΨ_M_ is depolarized (i.e., has a reduced ATP producing capacity) the mitochondria exerts control by inhibiting the local IP_3_-mediated Ca^2+^ signal so that the wave does not progress. Thus, as the Ca^2+^ signal regeneratively propagates from site to site through the cell (like a “fire-beacon” network), mitochondria act as “beacon-wardens” and determine whether or not the Ca^2+^ signal is transmitted to the next point of the cell 3,59. Presumably mitochondria modulate IP_3_R, but not voltage-dependent Ca^2+^ channel activity because the organelle is held close to the former, but not to the latter.

## Mechanisms and Driving Force for Mitochondrial Ca^2+^ Uptake

The large [Ca^2+^] range over which mitochondria are effective in regulating Ca^2+^ signaling prompted widespread interest into precisely how this is achieved. One mechanism is the MCU, a ruthenium red-sensitive, inwardly rectifying, and highly Ca^2+^ selective voltage-dependent channel of acknowledged importance in Ca^2+^ uptake by the organelle. In voltage-clamp experiments Ca^2+^ flux via MCU did not saturate until [Ca^2+^] exceeded 100 mM and the *K*_1/2_ was 19 mM 39. MCU may fulfill the low affinity role required of the organelle to modulate Ca^2+^ increases near Ca^2+^ channels (e.g., IP_3_R). Another proposed Ca^2+^ transporter present on the inner mitochondrial membrane is LetM1 33. LetM1, identified in genome-wide siRNA screen studies, is proposed to be a high affinity Ca^2+^/H^+^ exchanger that transports Ca^2+^ into mitochondria during low (<1 μM) global [Ca^2+^]_c_ increases when the free mitochondrial matrix [Ca^2+^] is also low (∼5 μM) 33. LetM1 may fulfill the role required for high affinity Ca^2+^ uptake by mitochondria. However, LetM1 was suggested to be a K^+^/H^+^ exchanger 56 several years before being proposed as a Ca^2+^ transporter. The Ca^2+^ transport facility attributed to LetM1 is not universally accepted 18,57.

Mitochondrial Ca^2+^ uptake mechanisms pass Ca^2+^ down an electrochemical gradient generated across the inner mitochondrial membrane by an outward movement of H^+^ via complexes I, III and IV of the electron transport chain. This proton movement establishes both the electrical potential difference (ΔΨ_M_) and the [H^+^] gradient. The [H^+^] gradient is significant; cytoplasmic pH is ∼7.2 while the pH within the mitochondrial matrix is ∼7.8. Indeed the effectiveness of complexes I, III, and IV in exporting H^+^ is emphasized by considering the average number of free protons within the matrix (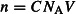; *n*, number of ions; *C*, concentration; *N*_A_, Avogadro's number; *V*, volume). A mitochondrial pH of 7.8 corresponds to a [H^+^] of ∼16 nM (1.58 E^−8^M). Taking the mitochondrion to be a prolate spheroid with dimensions of 2 μm (major axis) by 0.5 μm (minor axis) the volume (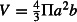 where *a* is the minor axis radius and *b* the major axis radius) is 0.26 fL. 1 g-H^+^/L = 6.023 E^23^ ions/L so that a [H^+^] concentration of 1.58 E^−8^ M = 9.5 E^15^ ions/L (1.58 E^−8^ × 6.023 E^23^) and the number of H^+^ per mitochondrion = 9.5 E^15^ × 0.26 E^−15^ *=* 2.5. Thus, on average there are only ∼2.5 H^+^ free within the mitochondrial matrix.

### Altering Mitochondrial Function and Ca^2+^ Signaling

The low internal proton numbers and significant pH gradient are critical for the performance of mitochondria and mitochondrial control of cell function. Together the transmembrane [H^+^] gradient and ΔΨ_M_ provide the protomotive force (approximately −180 mV) to drive ADP phosphorylation (catalyzed by the ATP synthase). ATP production approximately doubles with each 10 mV increase in protomotive force 37. The uptake of Ca^2+^ ions is driven by ΔΨ_M_. Unsurprisingly, a major method of determining the contribution of mitochondria to various cell activities (including Ca^2+^ signaling) is to collapse the proton gradient using drugs such as protonophores and electron transport chain inhibitors. Protonophores (e.g., CCCP and FCCP) are mildly acidic lipophilic compounds that are deprotonated in the mitochondrial matrix to form lipophilic anions. The deprotonated form crosses the inner mitochondrial membrane from the matrix, picks up a proton on the cytoplasmic side, and returns. In this way protonophores collapse the proton gradient and ΔΨ_M_ and, as a result, inhibit ATP synthesis and mitochondrial Ca^2+^ uptake. For example, protonophores slow the rate of [Ca^2+^]_c_ decline in smooth muscle (Figure 2) following depolarization-evoked Ca^2+^ entry. This experiment (Figure 2) reveals the ability of mitochondria to accumulate Ca^2+^, highlights the significance of the proton gradient in mitochondrial Ca^2+^ uptake and demonstrates the ease of use of protonophores to study mitochondrial activity.

However, protonophores may have significant off target effects and care is required in interpreting data from these experiments. Protonophores incorporate into the plasma membrane as well as the inner mitochondrial membrane and by facilitating the flux of protons may substantially alter the cytoplasmic pH. The effect of protonophores may be substantial. Extracellular pH is ∼7.4 (i.e., a [H^+^] of ∼40 nM) while cytoplasmic pH is ∼7.2 (i.e., a [H^+^] of ∼63 nM). The [H^+^] is thus highest in cytoplasm and lower in the extracellular space. However, the resting plasma membrane potential (approximately −60 mV; established by K^+^ permeability) may remain unaltered in the presence of protonophores. Because of its magnitude, the plasma membrane potential will determine the net flux of H^+^ and the concentration of H^+^ in the cytoplasm will increase via protonophore activity (i.e., decrease in pH). A 60 mV (inside negative) membrane potential difference will result in ∼10-fold increase in cytoplasmic [H^+^] to 400 nM (i.e., 10 times the external [H^+^]). Therefore, cytoplasmic pH will decrease from 7.2 to 6.4 when a protonophore is applied. Such a substantial decrease in pH is likely to exert several physiological changes and could result in a false-positive misinterpretation of the effects of protonophores on mitochondrial activity. A way around the pH change is to control cytoplasmic pH (in patch clamp experiments) using high concentrations of H^+^ buffers for example, 30 mM HEPES 12,13,49 or to target the protonophore specifically to the mitochondria to ensure significant cytoplasmic pH changes do not occur 11.

Even when changes in pH are considered and controlled, drugs which alter mitochondrial function may also alter the extent of free radical generation or ATP levels in cells (Table 1). Collapse of the proton gradient does not just prevent the production of ATP but results in the ATP synthase running in reverse to become an ATPase and deplete the cell of ATP, an effect prevented by using oligomycin in combination with drugs that collapse the proton gradient.

**Table 1:** Effect of drugs that alter mitochondrial function

Drug	Primary target	Off target/unwanted effects	Other limitations
Uncouplers/protonophores: CCCP, FCCP, DNP, 1799	ΔΨ_M_	Cytosolic pH changes; ATP consumption due to ATPsynthase reversal	Increased H^+^ permeability of other cellular membranes
Antimycin A	Complex III blockade (at Q_n/i_ site)		ROS production
Rotenone	Complex I blockade		Can cause ROS production, depending on substrate utilized
Myxothiazol	Complex III blockade (at Q_p/0_ site)		
Ruthenium red	MCU	Ca^2+^ and K^+^ channels	Low cell permeability
Ru360	MCU		Low cell permeability
CGP37157	mNCX	>10 μM also inhibits other Ca^2+^ channels	
Oligomycin	ATPsynthase		
Cyclosporin A	Permeability Transition Pore (PTP) by binding cyclophilin D in matrix	Immunosuppresant that also inhibits calcineurin by binding to cytosolic cyclophilins	
Atractyloside/carboxyactractyloside	Adenine Nucleotide Translocator (ANT) competitive inhibitor, locks ANT into cytosolic-facing conformation & promotes PTP opening		
Bongkrekic acid	Non-competitive inhibitor of ANT, locks ANT into matrix-facing conformation & inhibits PTP opening		
Ionomycin	Ca^2+^>Mg^2+^/2H^+^ exchange	ΔΨ_M_ depolarization when Ca^2+^ is present	
A23187	Ca^2+^/2H^+^ exchange	ΔΨ_M_ depolarization when Ca^2+^ is present	Fluorescence that interferes with fluorescent Ca^2+^ dyes (4-bromo-A23187 is non-fluorescent version)
Azide	ATP synthase inhibitor		
Cyanide	Complex IV inhibitor		

When each of these issues is considered and controlled, drugs which alter mitochondrial function provide a powerful experimental tool to examine the role of mitochondria in Ca^2+^ signaling.

### Measuring Mitochondrial Function

One of the most common methods of examining mitochondrial function is to optically measure the output of one of several fluorophores that are sensitive to ΔΨ_M_. The most popular belong to various families of compounds, which include rhodamine fluorophores 54, carbocyanins such as JC-1 66 and DiOC6 69, merocyanines 34 or oxonols 17. The major indicators presently used are the rhodamine fluorophores and JC-1 which will be discussed further below.

**Figure 6:**
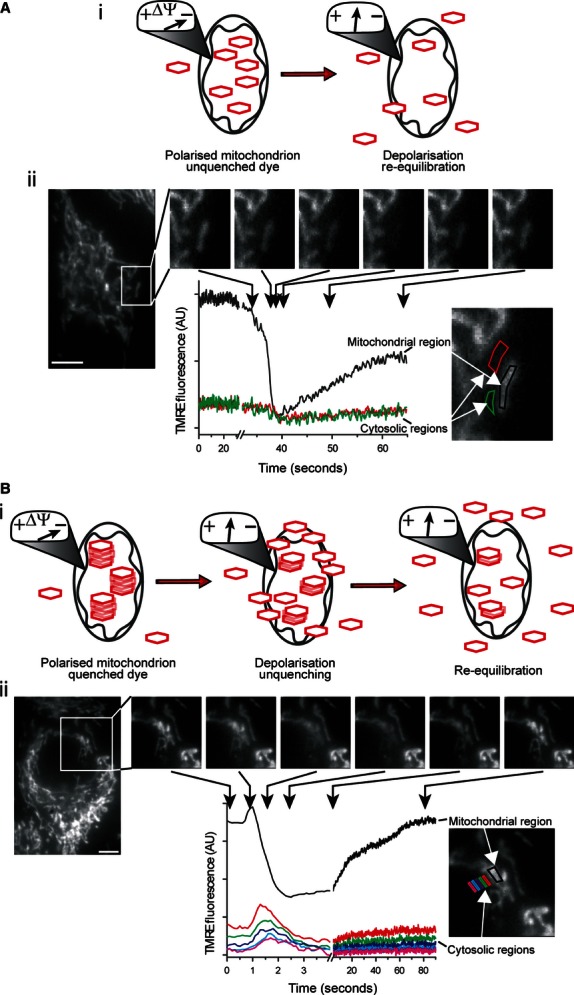
Membrane-permeant, lipophilic fluorescent cations can be used to monitor ΔΨ_M_ in “quenched” and “unquenched” modes. These fluorophores equilibrate across membranes in a Nernstian fashion (i.e., ∼10-fold per 60 mV). (**A**) at low fluorophore concentration the observed fluorescence is proportional to ΔΨ_M_ (i). A transient ΔΨ_M_ depolarization of an individual mitochondrion in a primary cerebral vascular smooth muscle cell loaded with TMRE (100 nM) resulted in a transient decrease in the fluorescence intensity of the mitochondrial region (ii, black line) with no measureable fluorescence change in the surrounding cytosolic regions (ii, red & green lines) (ii, scale bar = 5 μm), as visualized by epifluorescence microscopy; that is the change in [TMRE] outside the mitochondria, after ΔΨ_M_ depolarization, is presumably rapidly decreased in the larger volume of the cytoplasm so that a change in fluorescence is not measured. (**B**) At higher concentrations these fluorophores aggregate in mitochondria and self-quench, so that the observed fluorescence is no longer directly proportional to ΔΨ_M_. ΔΨ_M_ depolarization relieves this quenching, resulting in a transient *increase* in fluorescence (Bi & Bii). In this case, a transient ΔΨ_M_ depolarization of an individual mitochondrion resulted in a transient increase in the fluorescence intensity of the mitochondrial region (Bii, black line) and an observable dissipation of the released fluorophore into the surrounding cytosolic regions (Bii, colored lines) (ii, scale bar = 5 μm) because of the larger amount of fluorophore being released from mitochondria to the cytoplasm. When the dye is fully unquenched, the fluorescent signal declines as the dye continues to leave the mitochondria.

Rhodamine fluorophores (e.g., TMRE; TMRM; rhod-123) are fluorescent lipophilic cations that pass readily through lipid bilayers because their charge is dispersed over a large surface area. The mitochondria's negative membrane potential drives the fluorescent lipophilic cations into the matrix 2,68 and fluorophore distribution is described approximately by an Nernstian relationship, that is, ∼10-fold accumulation for every 61.5 mV of membrane potential at 37°C. Because of the large mitochondrial surface area to volume ratio, the accumulation of the fluorophore in the organelle changes rapidly (<1 s) with variations in the mitochondrial membrane potential 12. The more hyperpolarized (increasingly negative) the mitochondria become, the more fluorophore accumulates; the more depolarized (increasingly positive), the less fluorophore accumulates. When these fluorophores are used at low concentrations, changes in fluorescence signal follow changes in dye accumulation in mitochondria. Depolarized mitochondria will have a lower fluorophore concentration and a smaller fluorescence signal. Hyperpolarized mitochondria will have a higher fluorophore concentration and greater fluorescence signal (Figure 6A).

However, these fluorophores are somewhat complicated in their action and at high concentration form non-fluorescent aggregates (i.e., “quench”) so that fluorescence emission is reduced. Under these conditions, when mitochondria are hyperpolarized more fluorophore will enter the organelle, but this will cause additional quenching to *decrease* the fluorescent signal. Conversely, when mitochondria are depolarized, fluorophore concentration will decrease and fluorescence emission will now *increase* because the lower fluorophore concentration results in unquenching (Figure 6B). Under continued depolarization, the fluorophore will continue to leave the mitochondria. Eventually, when the fluorophore is unquenched, the fluorescent signal will decline with depolarization (Figure 6B).

The response from the rhodamine fluorophores therefore depends on the concentration at which they are used, which are referred to as either “quenching” (Figure 6B) or “unquenching” (Figure 6A) modes. The precise concentrations for quenching or unquenching modes should be determined empirically for each cell type. Typically <10 nM will result produce an unquenched concentration within the mitochondrial while >100 nM will result in quenching. There are advantages to each mode of fluorophore behavior. In unquenching mode, there is considerable sensitivity; changes in ΔΨ_M_ of a few millivolts may be detected and imaging ΔΨ_M_ of single mitochondria is possible. The signal may also be quantified in unquench mode. The relationship between fluorescence and ΔΨ_M_ is described from the ratio of the free mitochondrial fluorophore concentration to free cytosolic fluorophore concentration and is an exponential function of ΔΨ_M_ 41,61. However, in practice several difficulties limit quantification. First, the fluorophores may exhibit significant binding to the inner mitochondrial membrane so that most fluorescence comes not from free fluorophore, but from bound fluorophore 61,71. Changes in binding of the bound fluorophore are non-Nernstian and there is a larger accumulation than is predicted by the Nernst equation alone. While some studies have carefully addressed these issues to quantify ΔΨ_M_ 24,61, the majority of studies measure relative changes in fluorescence as a measure of ΔΨ_M_.

“Quench” mode has the advantage of producing larger signals (when compared with those in unquench mode) which are detected readily at a whole cell (rather than mitochondrial) level resulting in a more straightforward imaging procedure. The response of the fluorophore to membrane potential changes may be highly nonlinear in quench mode, however, and will show both increases and decreases in response to ΔΨ_M_ depolarization. Some consideration must also be given to the sampling frequency; transient changes in whole cell fluorescence in response to ΔΨ_M_ alterations may be missed with low frequency measurements.

As with all agents that are introduced into cells, fluorophores may have some toxicity to mitochondria and cells. Problems can arise from “phototoxicity” when fluorescent molecules react with molecular oxygen to produce free radicals that may damage subcellular components. The lowest possible excitation light intensity should be used. The fluorophores themselves at high concentrations may also inhibit the electron transport chain so the lowest possible fluorophore concentration should be also used.

Rhod-2 have also been used to measure mitochondrial [Ca^2+^] in native cells 22,23,49. However, rhod-2 is not straightforward to use and there is the potential for a significant cytosolic contribution to the signal. Rhod-2 AM ester is thought to be a suitable indicator for mitochondrial loading because it is the only cell-permeant Ca^2+^ indicator, which bears a net positive charge. This net positive charge is suggested to promote the uptake of rhod-2 AM into the mitochondrial matrix because of the strongly negative ΔΨ_M_. Once inside the mitochondrial matrix, esterases hydrolyse the AM group leaving the Ca^2+^-sensitive membrane impermeable form of rhod-2 trapped. However, while rhod-2 bears one net positive charge in the AM form, the de-esterfied rhod-2 bears three net negative charges and (even a partially de-esterified indicator) is unlikely to accumulate in mitochondria. The balance of esterase activity in the cytoplasm and mitochondria will therefore determine the major source of the signal from rhod-2 and is likely to contribute to variations in results among studies.

The other major class of fluorophore used commonly to measure the mitochondrial membrane potential is represented by JC-1. JC-1 is fluorescent and, like the rhodamine type fluorophores, accumulates in the mitochondria in a membrane potential dependent way. JC-1 has the useful feature of being a ratiometric indicator. In the cytoplasm JC-1 is usually “non-aggregated” (monomeric) and shows green fluorescence emission. When the concentration increases (in mitochondria) JC-1 aggregates and the fluorescence emission shifts from green to red. It is this fluorescence shift that permits ratiometric imaging of ΔΨ_M_^.^ In some studies JC-1 has been used successfully to measure ΔΨ_M_ 43,70, but other investigations have been less successful and report that fluorescence from the aggregated (red) form of the fluorophore may change in a way that is independent of ΔΨ_M_. Moreover, while the monomeric (green) form of JC-1 has been reported to equilibrate on a time scale similar to that of TMRM/TMRE, the *aggregate* (red) form of the dye takes six times longer to equilibrate 43. The aggregate form is required for JC-1 to act as a ratiometric probe and the differences in time required for equilibration of the two forms of the dye complicate interpretations of fluorescence changes. JC-1 may also report changes in ΔΨ_M_ when none exist, perhaps because of the dye's sensitivity to H_2_O_2_. Notwithstanding, JC-1 has been used successfully in some studies to image change in ΔΨ_M_.

One additional issue that requires consideration, regardless of the ΔΨ_M_ indicator, is the contribution that the plasma membrane potential makes to the signal measured from mitochondria, that is the fluorescence signal in the mitochondria is not independent of changes in plasma membrane potential. This situation arises because the concentration of fluorophore in mitochondria results from an equilibrium established by the *concentration in the cytoplasm* and the driving force for dye entry to mitochondria provided by ΔΨ_M_. Significantly, the concentration of fluorophore in the cytoplasm is itself an equilibrium involving the *extracellular* dye concentration and the *plasma membrane potential*. The contribution of the plasma membrane potential is significant and there is a 10-fold increase in cytoplasmic dye concentration for a 60 mV inside negative membrane potential. Drugs or physiological activators that change the plasma membrane potential will alter the concentration of fluorophore in the cytoplasm. As a result, there will be a re-equilibration of fluorophore concentration in mitochondria with the new cytoplasmic concentration and a change in mitochondrial fluorescence even in the absence of a ΔΨ_M_ change. This change in fluorescence could result in a misinterpretation of an effect of a drug or activator as altering ΔΨ_M_ when no such change has occurred. Solutions are either to clamp the plasma membrane potential or to correct the mitochondrial signals after measuring alterations in the plasma membrane potential 12,55,61.

Another significant consideration in the use of these drugs is an absence of spatial control of the organelles affected. One approach to overcome the lack of spatial control has been the development of photoactivatable forms of uncouplers 11. These photoactivatable uncouplers are membrane permeant, targeted to mitochondria, but are inactive until instructed otherwise by a locally directed pulse of light. Only those mitochondria exposed to the light will be affected by the drug. Individual cells or even single mitochondria can be targeted to generate precise spatial and temporal control. Photoactivateable drugs provide a significant advance in determining mitochondria's subcellular control of Ca^2+^ signals.

## Perspective

The mitochondrial proton gradient and ΔΨ_M_ drive Ca^2+^ uptake into the organelle. In studying the role of mitochondria in Ca^2+^ signaling, the proton gradient and ΔΨ_M_ are both measured and manipulated experimentally. Measurement of the mitochondrial electrochemical gradient is most often performed using membrane potential sensitive fluorophores. However, the signals arising from these fluorophores have a complex relationship with the mitochondrial electrochemical gradient and are altered by changes in plasma membrane potential. The solution to the latter problem is either to clamp the plasma membrane voltage or to determine the contribution of plasma membrane voltage changes to the mitochondrial signal. In determining the contribution of mitochondria to the cytosolic Ca^2+^ signal a large number of drugs offer a potent mechanism to alter the organelle's ability to take up Ca^2+^. These drugs alter mitochondrial Ca^2+^ uptake by changing the proton gradient across the inner mitochondrial membrane but may have significant off target effects. The off target effects include cytosolic pH changes, ATP depletion and alterations in free radical production. However, each off target effect may be controlled experimentally so the drugs offer a powerful method to study the role of mitochondria in Ca^2+^ signaling.

## Abbreviations

[Ca^2+^]: cytoplasmic Ca^2+^ concentration
AM: acetoxymethyl
CCCP: carbonyl cyanide m-chlorophenylhydrazone
CCh: carbachol
DiOC6: 3,3-dihexyloxacarbocyanine iodide
FCCP: carbonyl cyanide-*4*-(trifluoromethoxy)phenylhydrazone
IP_3_: inositol 1,4,5-trisphosphate
IP_3_R: inositol 1,4,5-trisphosphate receptor
JC-1: 5,5′,6,6′-tetrachloro-1,1′,3,3′-tetraethylbenzimidazolylcarbocyanine iodide
LetM1: leucine zippered-EF-hand containing transmembrane protein 1
mAChR3: muscarinic type 3 receptor
MCU: mitochondrial uniporter
mNCX: mitochondrial Na^+^/Ca^2+^ exchanger
PLC: phospholipase C
PTP: permeability transition pore
rhod-2: rhodamine-like fluorophores
RyR: ryanodine receptor
SR: sarcoplasmic reticulum
TMRE: tetramethylrhodamine ethyl ester
TMRM: tetramethylrhodamine methyl ester
ΔΨ_M_: mitochondrial membrane potential
